# Metabolic Endotoxemia Amplifies Estrogen Signaling Through Stromal Crosstalk in Obesity-Associated Breast Cancer

**DOI:** 10.3390/ijms27104338

**Published:** 2026-05-13

**Authors:** Emmy Drai, Daniela Nahmias Blank, Esther Hermano, Adi Yifrach, Ofer Chen, Ofra Maimon, Aron Popovtzer, Tamar Peretz, Amichay Meirovitz, Michael Elkin

**Affiliations:** 1 Department of Oncology, Hadassah Hebrew University Medical Center, The Hebrew University of Jerusalem, Jerusalem 91120, Israel; 2 Faculty of Medicine, The Hebrew University of Jerusalem, Jerusalem 91120, Israel; 3 The Legacy Heritage Oncology Center and Dr. Larry Norton Institute, Soroka University Medical Center, Faculty of Medicine, Ben Gurion University of the Negev, Be’er Sheva 84101, Israel

**Keywords:** breast carcinoma, obesity, estrogen, fibroblasts, macrophages

## Abstract

Obesity has been consistently associated with several types of malignant tumors, including hormone-responsive breast carcinoma (BC) in postmenopausal patients. BC is the most diagnosed malignancy and the leading cause of cancer-related morbidity and mortality among women worldwide. The predominant association between obesity and hormone-responsive BC subtypes is often explained by the increased local production of estrogens due to upregulation of aromatase, the rate-limiting enzyme in estrogen biosynthesis, in the chronically inflamed “obese” adipose tissue. However, the underlying molecular mechanisms are not fully defined. One potential mediator of inflammatory responses in the setting of obesity is bacterial endotoxin. Indeed, subclinical concentrations of endotoxin are chronically present in the circulation of obese patients and experimental animals (the phenomenon termed ‘metabolic endotoxemia’ [ME]). Here, we investigated whether ME conditions are mechanistically involved in augmented estrogen reactivity that sustains the obesity-BC link, focusing primarily on ME effects on key cellular components of the BC microenvironment (i.e., fibroblasts, macrophages). Our findings identify ME as a microenvironmental driver that directly induces aromatase expression in adipose fibroblasts, and simultaneously triggers upregulation of estrogen receptor levels in breast tumor cells via a macrophage-mediated mechanism. This dual action of ME in fueling obesity-accelerated hormone-dependent breast tumorigenesis may suggest new avenues for both therapeutic intervention and prevention of BC-promoting consequences of excess adiposity in an increasingly obese population.

## 1. Introduction

Metabolic endotoxemia (ME) is a condition characterized by the chronic presence of increased (yet subclinical) levels of bacterial endotoxin in the circulation of obese individuals and experimental models of obesity [1,2,3,4,5,6,7,8]. In ME, small quantities of endotoxin are translocated from the gut lumen to the circulation due to impaired intestinal permeability [6,9,10,11]. Importantly, adipose tissue is considered the primary site where endotoxin tends to accumulate and drive aberrant inflammatory responses [12,13]. ME has been proposed to underlie the mechanistic link between excess adiposity and several pathological conditions, e.g., insulin resistance, type 2 diabetes mellitus, pancreatitis, and cardiovascular complications [1,6,12,13,14,15,16]. Additionally, a possible contribution of ME in the pathogenesis of specific types of obesity-influenced malignancies was proposed by us [8] and others [17,18]. Indeed, obesity (defined as a body mass index [BMI] above or equal to 30 kg/m^2^) has been associated with at least 13 different types of malignant tumors (i.e., breast, esophageal, gastric, colorectal, hepatocellular and pancreatic carcinoma, multiple myeloma, gallbladder, endometrial, ovarian, renal and thyroid cancers) [19,20,21]. Endotoxin is a canonical activator of Toll-like receptor 4 (TLR4), which, in addition to its well-documented expression by immunocytes, was detected in tumor cells and implicated in tumorigenesis at several anatomic sites [17,22,23,24].

We previously demonstrated that ME enhances the malignant phenotype of TLR4-expressing breast carcinoma (BC) cells, and proposed ME as a possible mechanism underlying the breast tumor-promoting action of obesity [8,25]. Remarkably, in vivo experimental ME promotes the growth of tumors formed by E0771 BC cells [which are considered a luminal B subtype and express estrogen receptor (ER), although at relatively low levels [26,27,28,29]] to a greater extent than ER-negative MDA-MB-231 tumors (~2-fold increase, Supplementary Figure S1A, and ref. [8]). On the other hand, in vitro ME conditions (i.e., 0.1 ng/mL endotoxin, reflecting the levels reported in clinical/experimental obesity [1,2,3,4,5,6,10]) promote proliferation of E0771 and MDA-MB-231 cells to the same extent (Supplementary Figure S1B). These observations suggest an involvement of a hormone-dependent mechanism in the BC-promoting action of ME, as well as a contribution of the host-derived microenvironment. Consistent with the latter assumption, key cellular players in the obesity-associated BC-promoting microenvironment, i.e., macrophages and fibroblasts residing in the breast/adjacent adipose tissue [30,31,32,33,34,35], express TLR4 (Supplementary Figure S2) and are therefore capable of responding to subclinical levels of endotoxin typical for ME.

It is important to note in this respect that, unlike in cancer types linked to obesity, in BC, an obesity-related increase in tumor risk was observed only in ER+ subtypes and solely in postmenopausal patients [19,25,36,37,38,39,40,41,42,43,44,45]. This notion suggests that the obese state promotes ER+ BC primarily through hormone-dependent mechanisms. One suggested mechanism appears to involve abnormal activation of macrophages, which adopt a maladaptive phenotype that dictates changes in expression levels of ERα in normal or malignant mammary epithelial cells [46,47]. Even better recognized is the mechanism that involves increased local production of estrogen in chronically inflamed “obese” adipose tissue (primary site of estrogen production in postmenopausal women), due to induction of aromatase, the rate-limiting enzyme in estrogen biosynthesis. Importantly, in adipose tissue of postmenopausal women, aromatase is predominantly expressed in adipose stromal cells, i.e., fibroblasts, and is virtually absent from mature adipocytes [48]. Thus, adipose fibroblasts represent the principal cellular source of estrogen production after cessation of menstrual cycles [48,49,50,51,52]. In obesity, the hormone production is augmented, reportedly due to heterotypic interactions between immune and stromal compartments of adipose tissue [31,34,44,51,52,53,54]. Specifically, it was proposed that adipose/tumor tissue infiltrating macrophages, which become adversely activated under obese conditions, secrete inflammatory mediators (e.g., TNFα) capable of inducing expression of aromatase in adipose fibroblasts [33,49,50,52,55]. Thus, in obese postmenopausal patients, production of estrogen in adipose tissue (including breast adipose, which is in continuity with epithelial elements of the mammary gland [56]) can be markedly increased [57], promoting progression of ER+ BC. Nevertheless, the exact identity of molecules triggering the adverse responses of immune/stromal components in “obese” adipose tissue was not properly determined. Notably, in ME, endotoxin homes to adipose tissue [12,13] and, according to our hypothesis, can modulate abnormal responses of immune and stromal components in the setting of excess adiposity. In this study, we demonstrate that in obesity-associated BC, ME enhances ER-mediated tumor progression by orchestrating adverse interactions in BC microenvironment. Our present findings indicate that ME directly induces aromatase expression in fibroblasts, and simultaneously upregulates ER expression in BC cells in a macrophage-dependent manner. Collectively, these findings suggest that ME may establish a mechanistic bridge between obesity and hormone-dependent BC progression.

## 2. Results

**Figure 1:**
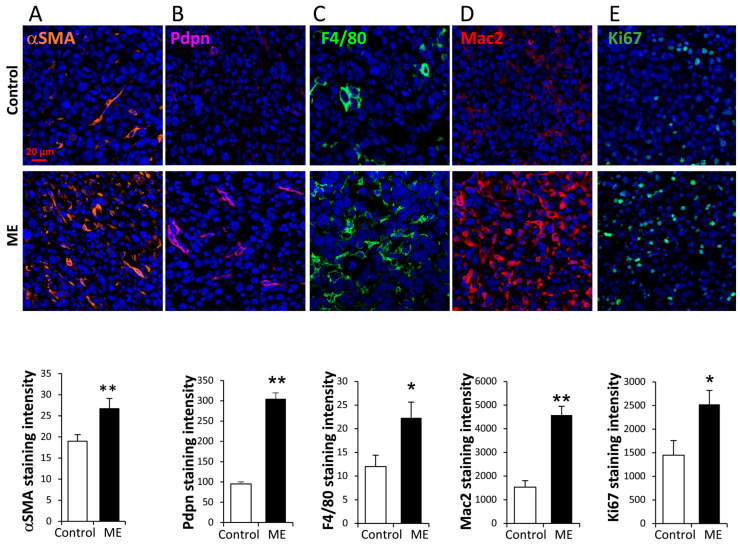
**Stromal and proliferation markers are increased in murine E0771 orthotopic breast tumors under chronic ME conditions.** C57/Bl6 mice were implanted sc. with the osmotic mini-pump (as described in Section 4) filled with either saline (**control**) or endotoxin (to infuse 300 microg per kg per day, resulting in plasma endotoxin concentrations corresponding to metabolic endotoxemia, **ME**). Ultrapure LPS from *E. coli* 0111:B4 (Invitrogen) was used to ensure the absence of contaminating TLR agonists, i.e., lipoproteins. Syngeneic BC cells E0771 were injected 3 days after pump implantation, as described in Section 4. Tumor tissue was harvested 15 days after cell injection. (**Top**) Immunofluorescent staining for αSMA (orange, (**A**)), podoplanin (Pdpn (purple, (**B**)), F4/80 (green, (**C**)), Mac2 (red, (**D**)), and Ki67 (olive green, (**E**)) of tumor tissue specimens harvested from saline-infused (**control**) and endotoxin-infused (**ME**) mice. Cell nuclei were counterstained with DRAQ5 (blue). (**Bottom**) Staining intensity was quantified using Zen software (Zen 3.4, blue version) per 0.07 mm^2^ microscopic field (all the fields chosen for analysis were located ≥1 mm from the tumor border), based on at least four sections from 3 mice per condition. Error bars represent ± SE. Two-sided Student’s *t* test * *p* < 0.05; ** *p* < 0.005.

**Increased accumulation of macrophages and fibroblasts in E0771 tumors under ME conditions.** As stated above, metabolic deregulation and obesity are known to promote BC through abnormal activation of the microenvironment in the tumor and adjacent adipose compartments [30,31,32]. In breast tissue (both healthy and tumorous), glandular epithelial elements are embedded in the adipose compartment, which consists of lipid-storing adipocytes, stromal cells and immunocytes [56]. Among those, fibroblasts (expressing aromatase [31,34,44,51,52,53,54]) and macrophages (producing tumor-promoting cytokines [33,49,50,52,55]) are recognized as key cell types promoting obesity-associated ER+ BC. Notably, endotoxin at subclinical levels typical for ME selectively triggers prolonged expression of moderate levels of cytokines in macrophages [58], including those implicated in obesity-associated BC progression, i.e., IL6 and TNFα. Based on the above data, we hypothesized that in the setting of excess adiposity, ME may fuel abnormal interplay between macrophage, fibroblast and adjacent adipose tissue compartments, accounting (at least in part) for the enhanced progression of hormone-responsive BC. To test this hypothesis, we first assessed the mobilization/activation of the cancer-associated fibroblasts and macrophages in tissue sections from E0771 tumors grown under ME conditions vs. tumors grown in control mice, applying an in vivo model of ME-associated breast cancer (as detailed in Section 4). Plasma endotoxin levels typical for ME (0.15 EU/mL) were measured (as described in Section 4) in the plasma of endotoxin-infused experimental mice at 14 days post-implantation, compared to undetectable levels (below the assay limit of detection, i.e., <0.005 EU/mL) in saline-infused control mice. The tumor tissues were then harvested and immunostained with antibodies directed against mouse macrophage-specific markers F4/80 [59] and Mac2 (AKA galectin-3, a member of the lectin family that drives alternative macrophage activation [60]), as well as cancer-associated fibroblast (CAF) markers αSMA and Pdpn (podoplanin, whose expression in CAF predicts unfavorable prognosis in ER+ BC patients [61,62]). As shown in Figure 1A,B, significantly increased numbers of αSMA- and Pdpn-positive CAF were detected in a ME-exposed tumor microenvironment. In parallel, a significant increase in macrophage infiltration was detected in the E0771 BC tumors exposed to experimental ME (Figure 1C,D), echoing a similar increase reported in obesity-associated E0771 tumors [63,64,65]. Moreover, applying immunostaining with an antibody directed against the proliferation marker Ki-67, we found that ME-induced activation of the tumor microenvironment was invariably associated with an increased proliferation rate of tumor cells (Figure 1E). These observations are in line with the ME-accelerated growth of E0771 tumors in vivo (Supplementary Figure S1), and point to the possible contribution of tumor microenvironment (i.e., fibroblasts and macrophages) to this phenomenon.

**Figure 2:**
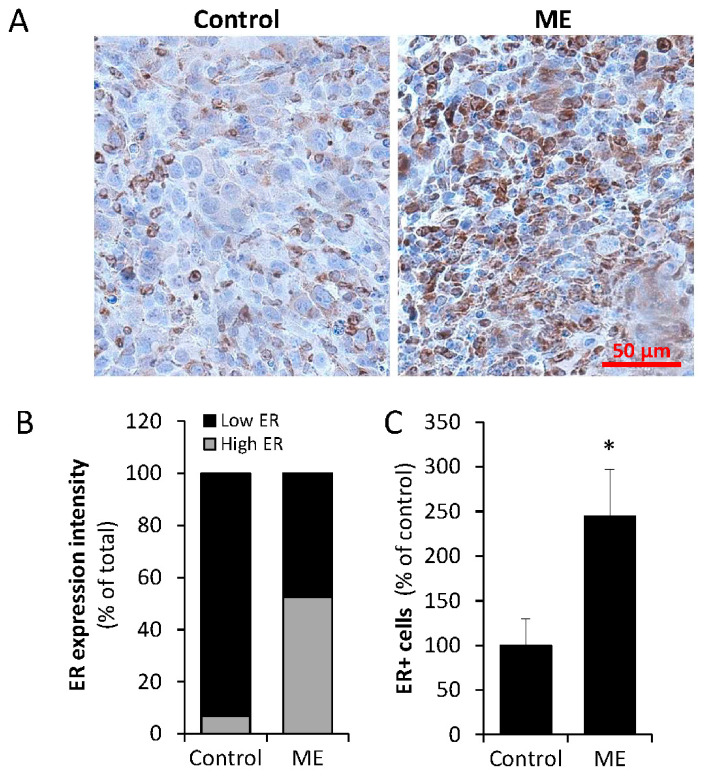
**Chronic ME increases ERα expression in E0771 orthotopic tumors in vivo.** Chronic ME was established as described in Section 4. Syngeneic BC cells E0771 were injected 3 days after pump implantation. Tumor tissue was harvested 15 days post cell injection and processed for immunohistochemistry, as described in Section 4. (**A**) Representative images of immunohistochemical staining (brown) with anti-ERα antibody in tumor tissue specimens derived from saline-infused (**control**) and endotoxin-infused (**ME**) mice. (**B**) The intensity of ERα staining in tumor tissue specimens derived from saline-infused (**control**) and endotoxin-infused (**ME**) was scored as weak (1), moderate (2), or strong (3), as described in Section 4; tumors with staining score 1 were categorized as “low ER” (black bars); tumors with staining score 2 and 3 as “high ER” (grey bars). Chi-squared analysis was then used to assess the relationship between ME conditions in vivo and high versus low ER levels in E0771 orthotopic tumors. Significant correlation between ME and high ER levels was detected: ~5-fold higher proportion of “high ER” tumors was noted under ME conditions versus control group (52% vs. 10%, chi-square test * *p* = 0.0041). A minimum of 3 microscopic fields per mouse/3 mice per condition were scored. (**C**) Percentage of ERα positive cells in tumor tissue specimens derived from saline-infused (**control**) and endotoxin-infused (**ME**) was determined as described in Section 4. A minimum of 100 tumor cells per tissue section were scored. Data shown are the percentage of ERα positive cells relative to control. Error bars represent ± Sdev. Two-sided Student’s *t* test * *p* < 0.001.

**Figure 3:**
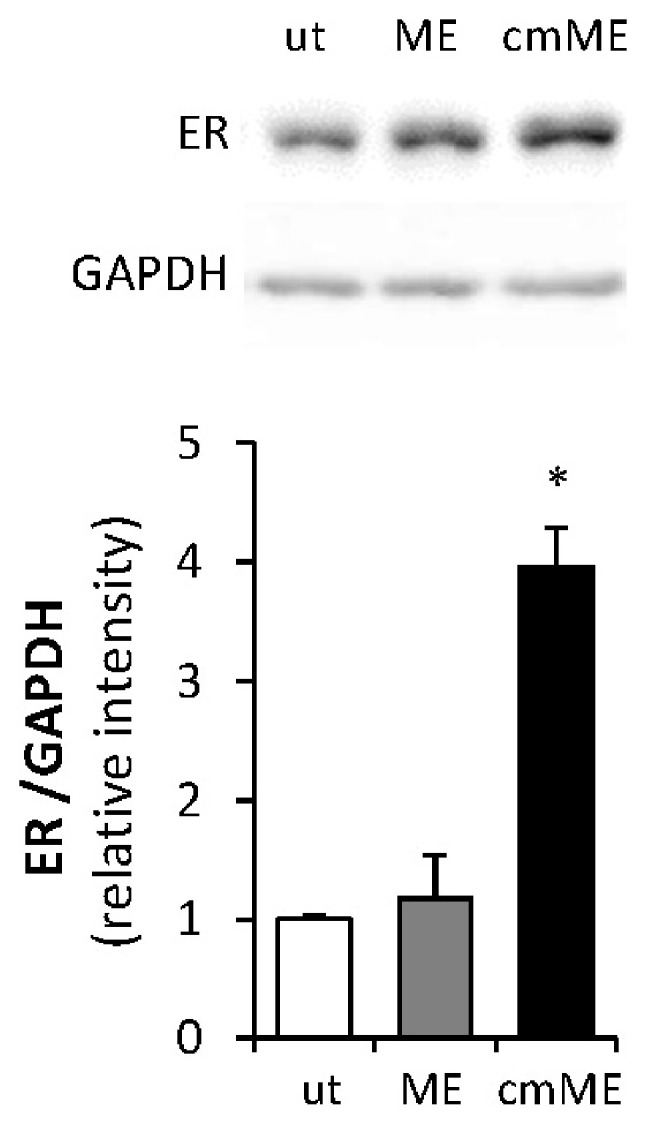
E0771 cells were either left untreated (**ut**) or incubated (24 h, 37 °C) with 0.1 ng/mL of endotoxin (concentration reflecting metabolic endotoxemia, **ME**), or with medium conditioned by macrophages stimulated by 0.1 ng/mL endotoxin (**cmME**). ERα protein levels were determined by immunoblotting (**top**) and quantified using ImageJ 1.54g software (**bottom**). Intensity ratio for ER/GAPDH is shown. The data are representative of at least 3 independent experiments; error bars represent ± SE.* *p* < 0.007.

**ME upregulates ERα expression levels**. As stated above, upon acquiring a maladaptive phenotype, macrophages are capable of inducing expression of ERα in estrogen-responsive malignancies (i.e., breast, endometrial cancer [47,66]). Given the cytokine-driven mechanism underlying this effect [66] and the ability of endotoxin levels reported in ME to activate macrophages toward a low-grade inflammatory phenotype [58], we next tested whether experimental ME affects ERα levels in E0771 tumors in vivo. Immunohistochemical examination of ERα expression levels in the tumor tissue derived from mice exposed to experimental ME vs. control mice revealed that expression of the ERα protein was significantly increased in E0771 tumors growing in mice under ME conditions (Figure 2A). Quantitation of ERα expression intensity (Figure 2B) and percentage of ER+ cells (Figure 2C) in the tumor tissue samples, performed as described in [67], revealed a significant increase in both parameters in the tumors derived from ME-exposed vs. control mice. Importantly, these in vivo findings were in agreement with observations made under conditions mimicking ME in vitro, i.e., incubation of E0771 cells with medium conditioned by macrophages that were either unstimulated or stimulated by 0.1 ng/mL of endotoxin. As shown in Figure 3, medium conditioned by macrophages incubated under ME-like conditions markedly upregulated ERα protein levels in E0771 cells.

**Figure 4:**
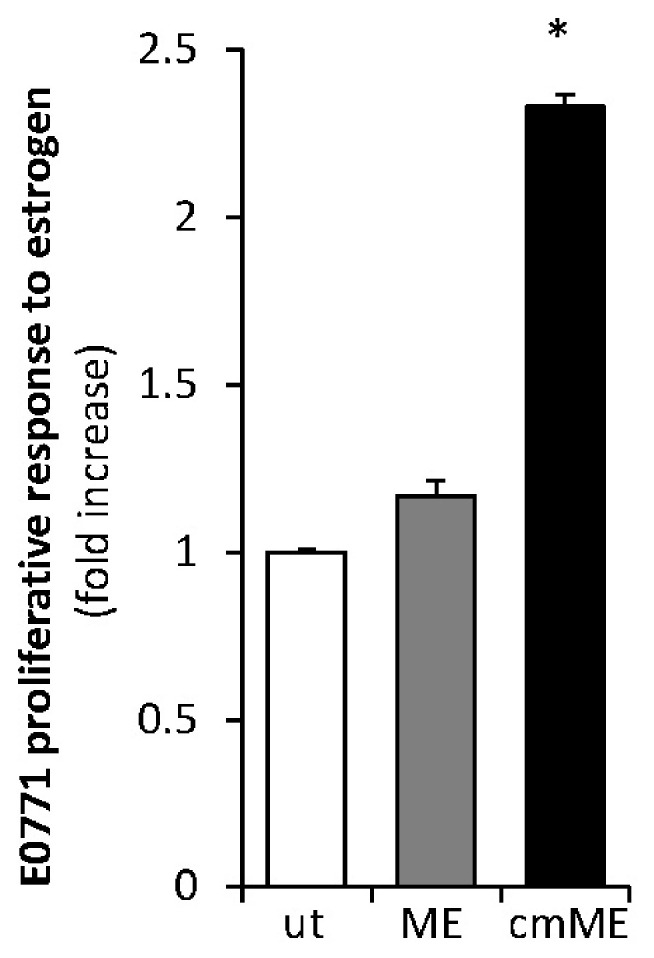
**Effect of medium conditioned by macrophages stimulated by ME conditions on estrogen sensitivity of E0771 cells.** Prior to estrogen treatment, E0771 cells were hormone-depleted by culturing for 2 weeks in phenol red-free medium supplemented with charcoal-stripped FCS (5%). Then the cells remained either untreated (ut) or were incubated with endotoxin (at a concentration of 0.1 ng/mL, corresponding to metabolic endotoxemia conditions, **ME**), or with medium conditioned by macrophages stimulated by 0.1 ng/mL endotoxin (**cmME**), and grown in the absence or presence of 10^−9^ M estradiol (96 h, 37 °C). Cell proliferation was analyzed by MTS assay. Data shown are the fold increase in cell proliferation in response to estradiol. * *p* = 0.027, Error bars represent ± SE.

**Figure 5:**
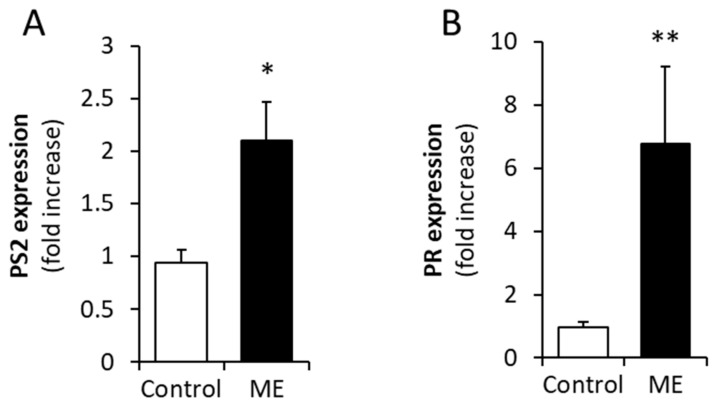
**ME increases the expression of estrogen target genes in E0771 orthotopic tumors.** Expression of the genes encoding pS2 (**A**) and progesterone receptor (PR, (**B**)) in E0771 tumors growing in saline-infused (**control**) and endotoxin-infused (**ME**) mice was assessed by qRT-PCR (*n* ≥ 3 per condition, * *p* = 0.004; ** *p* = 0.017).

**Macrophages under ME conditions confer estrogen sensitivity to E0771 cells.** Next, to validate the functional significance of ERα upregulation mediated by ME-stimulated macrophages, we examined the effect of medium conditioned by primary murine macrophages exposed to ME conditions on the proliferative response of E0771 cells to estrogen. Prior to the hormone treatment, the E0771 cells were depleted of estrogen for 2 weeks (as described in Section 4) and then treated with 17β estradiol in the presence or absence of medium conditioned by macrophages (which were either untreated or incubated with 0.1 ng/mL endotoxin). As shown in Figure 4, the presence of medium conditioned by endotoxin-stimulated macrophages significantly augmented proliferation of E0771 cells in response to estradiol, consistent with the upregulation of ERα levels depicted in Figure 3. In further agreement with the above observation, we detected significantly increased expression levels of mRNA encoding progesterone receptor and pS2 (two estrogen-regulated genes commonly used as indicators of augmented estrogen signaling) in E0771 breast tumors growing in ME-exposed vs. control mice (Figure 5). Collectively, these findings indicate that ME conditions can trigger macrophage-driven upregulation of ERα levels in BC cells, thus enhancing the proliferative response of the tumor to estrogen.

**Figure 6:**
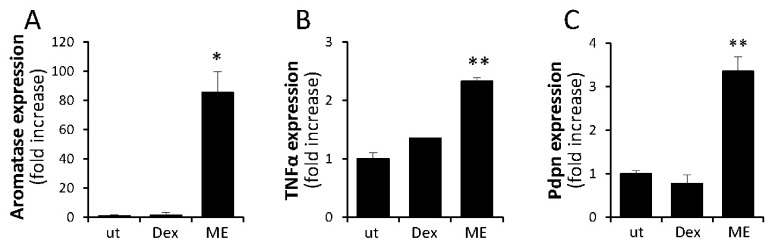
**Adipose fibroblasts upregulate aromatase, TNFα, and Pdpn expression in response to ME conditions in vitro.** Human mammary fibroblasts (HMFs), isolated from breast adipose tissue of healthy women, remained untreated (**ut**) or incubated with dexamethasone (**DEX**, 250 nM, grey bar), alone or in the presence of 0.1 ng/mL of endotoxin (**ME**). Quantitative real-time RT-PCR was used to assess the expression of human aromatase (**A**), TNFα (**B**) and Pdpn (**C**) RNA levels. * *p* < 0.02; ** *p* < 0.005.

**ME directly induces aromatase in adipose fibroblasts.** In addition to ERα expression levels, augmented production of estrogen is known to profoundly affect obesity-influenced breast tumorigenesis and BC response to hormonal therapy [44,52]. Moreover, the BC-promoting action of obesity/metabolic dysregulation in postmenopausal patients is most often attributed to increased production of the hormone in ‘obese’ adipose tissue due to induction of aromatase, the rate-limiting enzyme in biosynthesis of estrogens [33,44,55,68]. Prior to menopause, aromatase is expressed in the ovary, the principal production site of estrogens in pre-menopausal women [57]. After menopause, the expression of aromatase and biosynthesis of estrogen takes place in extragonadal sites (although at a lower rate [57,69])—primarily in peripheral adipose, i.e., mammary and visceral adipose tissues [34,44,48,51,52,54]. Importantly, under metabolic deregulation/obese conditions, extragonadal production of estrogens is markedly increased [70,71,72,73], due to upregulation of aromatase expression in fibroblasts residing in “obese” adipose tissue [48,51,52,54,57,74]. Obesity-related upregulation of aromatase in fibroblasts is believed to be driven by macrophages, which are adversely activated under metabolic deregulation (via a TLR4-dependent mechanism) and secrete a cocktail of inflammatory modulators capable of inducing aromatase expression in fibroblasts [34,35,49,50,51,54,64,75,76]. We therefore assumed that ME conditions in the obese state contribute to adverse macrophage stimulation and consequent induction of aromatase expression in adipose tissue fibroblasts. To investigate the proposed mode of action, we used primary human mammary fibroblasts (HMFs) isolated from breast adipose tissue of healthy women undergoing reduction mammoplasty (as described in Section 4), a reliable model to study regulation of aromatase expression in adipose tissue [49,50,52]. However, contrary to our expectations, medium conditioned by macrophages stimulated by endotoxin at a ME-relevant concentration (i.e., 0.1 ng/mL) had no significant effect on aromatase expression by HMFs. We then noted that fibroblasts per se express TLR4 at levels comparable to macrophages (Supplementary Figure S2, also see refs. [77,78]) and, when stimulated by pro-inflammatory signals, i.e., bacterial products, are capable of generating cytokines [78,79] [including TNFα, known to activate the promoter of the *Cyp19a1* gene encoding aromatase [35,49,54]]. Hence, we hypothesized that ME conditions can directly affect aromatase expression in fibroblasts. To test this hypothesis, the HMFs were left untreated or incubated with 0.1 ng/mL endotoxin, mimicking ME. As shown in Figure 6A, a significant increase in aromatase expression was detected in HMFs in the presence of endotoxin. Since both IL-6 and TNFα were shown to induce aromatase in adipose tissue fibroblasts [35,49], we next assessed changes in the expression of these cytokines by HMFs in response to endotoxin. We found that TNFα (known to upregulate aromatase expression via stimulation of c-fos/c-jun binding to the AP-1 binding site upstream of promoter 1.4 of the *CYP19* gene, encoding aromatase [49]) was the only cytokine whose expression was increased in HMFs under ME conditions in vitro (Figure 6B). As shown in Figure 6C, ME conditions in vitro also resulted in a marked increase in the expression of Pdpn, a fibroblast marker associated with worse clinical outcomes in BC [61,62]. This observation is consistent with the previously reported ability of TNFα to upregulate Pdpn expression in human fibroblasts in several tissue types other than breast adipose [80,81], as well as with an increased number of Pdpn-positive CAF detected in E0771 tumors under chronic ME (Figure 1B). To our knowledge, this is the first demonstration that ME conditions directly induce TNFα and aromatase in breast adipose fibroblasts.

**Figure 7:**
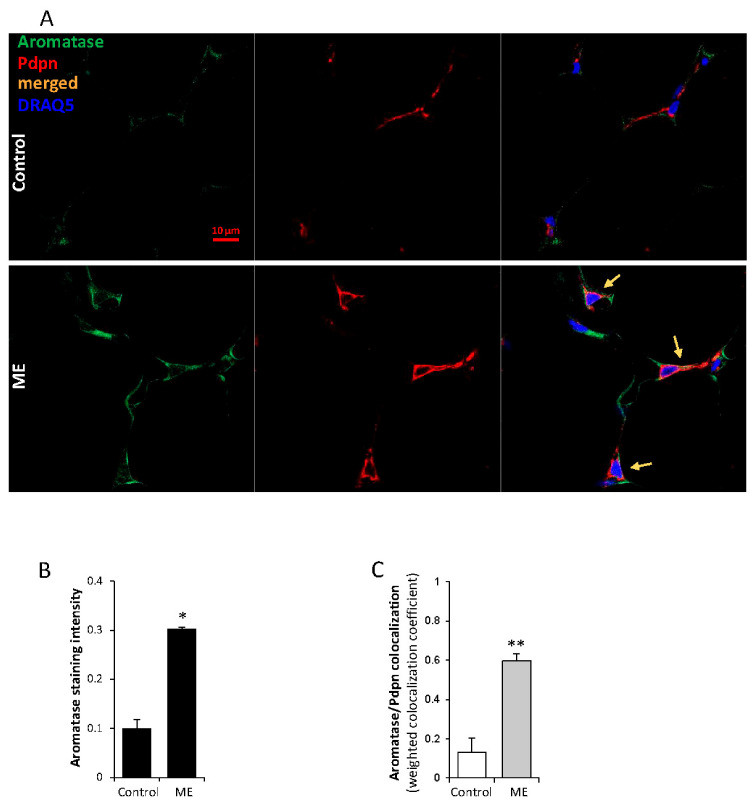
**Effect of chronic ME on aromatase expression by adipose fibroblasts in vivo.** (**A**) Sections of mouse adipose tissue samples derived from saline-infused (**control**) and endotoxin-infused (**ME**) mice (*n* ≥ 3) were harvested 18 days after pump implantation and processed for double immunofluorescent analysis using anti-aromatase (green) and anti-**Pdpn** (red) antibodies. Yellow arrows indicate aromatase-expressing **Pdpn**^+^ fibroblasts. Cell nuclei were counterstained with DRAQ5 (blue). Photographs are representative of **control** (upper panels) and **ME** (lower panels) samples. Scale bar = 10 μm. (**B**) Staining intensity of aromatase (**B**) and **Pdpn** was quantified based on at least four sections from 3 mice per condition using Zen software (Carl Zeiss) per 0.01 mm^2^ microscopic field. (**C**) Quantification of the degree of association between aromatase and Pdpn staining in mouse adipose derived from **control (empty bar)** and endotoxin-infused (**ME, filled bar**) mice (*n* ≥ 3) was performed using colocalization tool of Zen software. Bar graph shows the weighted correlation coefficient values for aromatase/Pdpn colocalization. Data are the mean ± SE; two-sided Student’s *t* test * *p* < 0.01; ** *p* < 0.002.

To better place these observations in the context of ME in vivo, we utilized sections of adipose tissue adjacent to E0771 orthotopic tumors growing in mice with chronic ME or control mice, and analyzed changes in fibroblast-derived aromatase expression under ME. As shown in Figure 7A,B, increased aromatase levels were detected by immunofluorescent staining in adipose tissue under chronic ME conditions. Consistent with the notion that fibroblasts represent the chief cellular source of aromatase in adipose tissue [48,51,52,54,57,74,82], double immunofluorescent staining for Pdpn and aromatase, along with confocal microscopy analysis (Figure 7C), detected a marked increase in aromatase-expressing Pdpn-positive (Pdpn^+^) adipose tissue fibroblasts in adipose tissue harvested from mice exposed to chronic ME, compared with control mice. In further agreement with fibroblast aromatase induction in vivo (Figure 7) and in vitro observation depicted in Figure 6B, a significant increase in TNFα^+^/Pdpn^+^ fibroblasts, concomitant with augmented TNFα levels, was detected in adipose tissue derived from mice with chronic ME (Supplementary Figure S3). Collectively, these observations support the notion that ME conditions trigger an autocrine TNFα loop in adipose fibroblasts, directly leading to aromatase upregulation (most likely, via AP-1-dependent activation [49] of the *CYP19* gene promoter).

## 3. Discussion

Cancer-promoting effects exerted by obesity and associated metabolic derangements are certainly multifactorial in all obesity-influenced tumor types [83,84]. Elevated circulating levels of insulin, glucose, inflammatory mediators, and misbalanced production of adipokines are the most extensively studied factors believed to contribute to cancer progression under an obese state [68,83,85,86,87]. Among obesity-associated malignancies, BC is unique in that the increase in tumor risk was largely confined to ER+ subtypes in postmenopausal patients [25,40,41,42,43,44,45], suggesting a critical role of estrogen signaling in the obesity-BC link. This pattern was traditionally attributed to excessive estrogen exposure, due to augmented extragonadal production of the hormone in chronically inflamed adipose tissue of obese women (in the face of cessation of the menstrual cycle) [33,55,68]. Augmentation of estrogen synthesis is often described within the context of adverse activation of macrophages, which, in turn, upregulate expression of estrogen-synthesizing aromatase in adipose tissue fibroblasts [34,35,49,50,51,54,64,75,76].

We have previously demonstrated that obesity and ME directly affect the growth and malignant phenotype of BC cells expressing the TLR4 receptor [8]. Notably, direct exposure to ME-like conditions in vitro increased proliferation similarly in ER+ and ER− BC cells, whereas in vivo ME preferentially accelerated ER+ tumor growth (Supplementary Figure S1), pointing to microenvironment-dependent mechanisms. In the present study, we identify ME as a microenvironmental driver of two “arms” of estrogen signaling: ME directly induces aromatase in adipose fibroblasts (Figure 6A and Figure 7) and, in parallel, triggers macrophage-mediated ERα upregulation in breast tumor cells in vitro (Figure 3, Figure 4 and Figure 5) and in vivo (Figure 2).

A central finding of this study is that ME conditions can directly activate breast adipose fibroblasts to upregulate TNFα and aromatase in a macrophage-independent manner. Using primary human mammary adipose fibroblasts and adipose tissue adjacent to E0771 tumors in vivo, we show that ME conditions induce an autocrine TNFα loop in fibroblasts, leading to increased aromatase expression and expansion of a Pdpn-positive aromatase-expressing fibroblast population in adipose tissue (Figure 6 and Figure 7 and Figure S3). To our knowledge, this is the first demonstration that endotoxin at obesity-relevant subclinical levels directly induces aromatase and TNFα in breast adipose fibroblasts, thereby providing a mechanistic link between ME and extragonadal estrogen biosynthesis.

In parallel, the present study extends the previous observation that BC-associated macrophages can affect ER expression levels in tumor cells. Previous reports have implicated tumor-infiltrating immunocytes (including macrophages) in the regulation of steroid hormone receptor expression in several hormone-responsive cancer types, including BC [47,66,88,89]. Here, we demonstrate that ME conditions in vivo increase ERα expression and the fraction of ER+ cells in E0771 tumors, and macrophage-conditioned medium generated under ME conditions induces ERα protein levels, estrogen-dependent proliferation, and expression of ER target genes (PR, pS2) in BC cells (Figure 2, Figure 3, Figure 4 and Figure 5).

Collectively, our data support a hypothesized role of ME in mediating the BC-promoting action of obesity at the microenvironmental level, i.e., by priming both fibroblasts and macrophages, leading to increased estrogen production and heightened ER signaling in ER+ tumor cells. Moreover, these fibroblast- and macrophage-directed effects may explain why ME “favors” ER+ tumors in vivo, despite similar proliferative effects on ER+ and ER− cell lines in vitro. In ME-exposed mice, fibroblast aromatase induction amplifies local estrogen availability, while macrophage-mediated ERα upregulation increases tumor cell sensitivity even to very low hormone concentrations. This dual modulation of estrogen synthesis and receptor abundance may be particularly relevant in postmenopausal women, where adipose fibroblasts are the principal estrogen source and systemic estrogen levels are low. Given the occurrence of ME in obese patients [2,5,7,8] and the ability of the endotoxin to home to adipose tissue [13], such a mechanism provides a conceivable cellular explanation for the selective enrichment of obesity-accelerated disease in ER+ BC and may contribute (at least in part) to the diminished efficacy of aromatase inhibitors in obese BC patients [90,91,92,93].

Several limitations of our study should be acknowledged. Our in vivo experiments relied primarily on the E0771 murine BC model, which currently represents the only syngeneic ER+ breast cancer cell line available for interrogating obesity/ME-accelerated hormone-dependent tumorigenesis in immunocompetent mice. This constrains the generalization of our findings to other ER+ subtypes and to the clinical heterogeneity of human disease. An additional limitation is that, while our data implicate TLR4 signaling and TNFα in mediating ME effects on fibroblasts and macrophages, no inhibitory/loss-of-function studies (e.g., TLR4 knockout, pharmacologic inhibition, or TNFα neutralization) have been performed. Specifically, confirmation of the role of TNFα as a clinically actionable target, and its translational validation, are important directions for future investigation. Such studies are expected to clarify whether pharmacological interruption of the ME-TNFα-aromatase axis (i.e., by TNFα blockade) could improve aromatase inhibitor efficacy in the obese patient population. Lastly, our work does not fully address potential interactions with other obesity-related factors (e.g., hyperinsulinemia, hyperglycemia, or adipokine imbalance). Thus, future studies integrating these factors, as well as prospective corroboration in a clinical setting, are required to fully validate ME in BC patients as a biomarker of endocrine therapy response and as a potential target for preventing/mitigating hormone-dependent BC progression in obese postmenopausal patients.

## 4. Materials and Methods

**Orthotopic immunocompetent murine model of obesity-associated breast cancer.** The mouse model of obesity-associated BC growth was established essentially as described [64]. Briefly, ten-week-old female C57BL/6J mice (Envigo, Jerusalem, Israel) were fed ad libitum a high-fat diet (HFD) (Teklad TD.06414, 60% of total calories from fat), or a control diet (CD) (Teklad 2018S) for 15 consecutive weeks. On experimental week 12, when HFD-fed animals became obese, E0771 cells were injected orthotopically into the 4th left mammary fat pads of both HFD-fed (obese) and control diet-fed (lean) mice (5 × 10^5^ cells per injection). The tumor volume was monitored until experimental week 15. Animals were then sacrificed, tumor tissue samples collected and snap-frozen for protein extraction.

**Murine model of ME-associated breast cancer.** The mouse model of ME was established essentially as described [1]. Briefly, nude or syngeneic C57BL/6 mice were implanted subcutaneously with the micro-osmotic pumps (Alzet, Cupertino, CA, USA; Model 1004), filled with saline (control) or with endotoxin (ultrapure LPS from *E. coli* 0111:B4, Invitrogen), to infuse 300 μg × Kg^−1^ × Day^−1^ (these conditions produce plasma LPS concentrations similar to those found in ME [1]). Then, 3 days later, when the endotoxin infusion rate reaches a constant level and remains stable for 28 days [94], murine E0771 or human MDA-MB-231 BC cells (5 × 10^5^ cells per injection) were injected orthotopically into the 4th left mammary fat pad of C57BL/6 or nude mice, respectively. Tumor growth was monitored for two weeks. Mice were then sacrificed, tumor tissue samples collected and snap-frozen for protein extraction. Before sacrifice, the persistence of ME in the circulation of mice implanted with endotoxin-infusing (but not saline-filled) pumps was validated by determination of endotoxin levels in mouse plasma samples collected 14 days post pump implantation, essentially as described in [8], using the chromogenic kinetic Limulus Amoebocyte Lysate assay, applying Endosafe-nextgen portable test system apparatus, multi-cartridge system readers and cartridges with a lower limit of detection of 0.005 EU/mL endotoxin (Charles River, Charleston, SC, USA).

**Macrophage isolation and treatment.** Primary mouse macrophages were obtained by applying the isolation procedure described in [64,95,96]. Some cells were treated with 0.1 ng/mL of ultrapure endotoxin derived from *E. coli* 0111:B4 (Invitrogen, Waltham, MA, USA), as indicated in Section 2.

**Fibroblast isolation and treatment.** Human fibroblasts isolated from normal mammary adipose tissue of women undergoing reduction mammoplasty (as described in [97]), were kindly provided by Dr. A. Orimo (Juntendo University of Medicine, Tokyo, Japan) and grown in DMEM medium supplemented with 1 mM glutamine, 50 μg/mL streptomycin, 50 U/mL penicillin and 10% fetal calf serum (Biological Industries, Beit-Haemek, Israel) at 37 °C and 8% CO_2_. Mouse fibroblasts, isolated from normal mammary adipose tissue of 10-week-old female C57BL/6 mice as described in [98], were grown in RPMI medium supplemented with 1 mM glutamine, 50 μg/mL streptomycin, 50 U/mL penicillin and 10% fetal calf serum (Biological Industries) at 37 °C and 8% CO_2_. Prior to treatment with endotoxin, cells were kept for 24 h in serum-free medium and then either left untreated or incubated with 250 nM dexamethasone (known to allow for aromatase expression in human fibroblasts [49]), alone or in the presence of 0.1 ng/mL of endotoxin, as indicated in Section 2.

**Breast carcinoma cell culture.** E0771 cells were cultured in RPMI medium; MDA-MB-231 cells were cultured in DMEM supplemented with 1 mM glutamine, 50 µg/mL streptomycin, 50 U/mL penicillin and 10% fetal calf serum (Biological Industries) at 37 °C and 8% CO_2_. Some cells were treated with 0.1 ng/mL of endotoxin. For estrogen depletion, cells were cultured for 14 days in phenol red-free DMEM supplemented with 5% charcoal-stripped fetal calf serum (Biological Industries), antibiotics, and supplemented with l mM glutamine, prior to estrogen treatment.

**MTS Assay.** Cells were seeded in 96-well culture plates in phenol red-free DMEM, supplemented with 5% charcoal-stripped FCS. MTS assay (Promega, Madison, WI, USA) was performed according to the manufacturer’s instructions. Each experiment was performed at least 3 times. Each data point shows the mean of pentaplicate cultures.

**Antibodies.** Immunoblot analysis or immunohistochemistry was carried out with the following antibodies: anti–ERα (ab3575, Abcam, Cambridge, UK), anti–αSMA (ab124964, Abcam), anti-Pdpn (AF3244, R&D Systems, Minneapolis, MN, USA), anti-F4/80 (MCA497R, Serotec, Kidlington, Oxfordshire, UK), anti-Mac2 (CL8942AP, Cedarlane, Burlington, ON, Canada), anti-Ki67 (SP6, Thermo Fisher Scientific, Waltham, MA, USA), anti-aromatase (NB100-1596, Novus Biologicals, Centennial, CO, USA), anti-TNFα (AF-410-SP, R&D Systems), anti-Actin (C-2) (sc-8432, Santa Cruz, Dallas, TX, USA) and anti-GAPDH (EnCor Biotechnology Inc., Gainesville, FL, USA).

**Immunoblotting.** Cell and tumor tissue lysates were homogenized in lysis buffer containing 0.6% SDS, 1 mM Tris-HCl, pH 7.5, supplemented with a mixture of protease inhibitors (Roche, Basel, Switzerland) and phosphatase inhibitors (Thermo Scientific, Waltham, MA, USA). Equal protein aliquots were subjected to SDS-PAGE (10% acrylamide) under reducing conditions and proteins were transferred to a polyvinylidene difluoride membrane (Millipore, Darmstadt, Germany). Membranes were blocked with 3% BSA for 1 h at room temperature and probed with the appropriate antibody, followed by horseradish peroxidase-conjugated secondary antibody (KPL, Milford, MA, USA) and a chemiluminescent substrate (Biological Industries, Beit-Haemek, Israel). Band intensity was quantified by densitometry analysis using ImageJ software.

**Immunohistochemistry.** Paraffin-embedded slides were deparaffinized and incubated in 3% H_2_O_2_. Antigen retrieval was carried out by heating (20 min) in a microwave oven in citrate buffer. Slides were incubated with primary antibodies diluted in CAS-Block (Invitrogen) or with CAS-Block alone, as a control. Appropriate secondary antibodies (Nichirei Biosciences Inc, Tokyo, Japan) were then added and slides incubated at room temperature for 30 min. Color was developed using the DAB substrate kit (Thermo Scientific), followed by counterstaining with Mayer’s hematoxylin. Staining with the control IgG or without addition of primary antibody showed low or no background staining in all cases. Immunodetection of ERα in E0771 tumor specimens was performed using Abcam (ab3575) ERα antibody. Staining intensity [scored as weak (=1), moderate (=2), or strong (=3)] and percentage of positively stained tumor cells were determined in accordance with [67,99].

**Immunofluorescence.** For immunofluorescence analysis, Alexa-555 donkey anti-goat (Abcam) and CyTM2 donkey anti-rabbit (The Jackson Laboratory, ME, USA) antibodies were used as secondary antibodies. Nuclear staining was performed with 4′,6-diamidino-2-phenylindole (DAPI). Images were captured using a Zeiss LSM 5 confocal microscope (Zeiss, Oberkochen, Germany) and analyzed with Zen software (Zen 3.4, blue version, Carl Zeiss).

**Analysis of gene expression by quantitative real-time PCR (qRT-PCR).** Total RNA was isolated from cultured cells or snap-frozen tumor tissue samples using TRIzol (Invitrogen, Waltham, MA, USA), according to the manufacturer’s instructions, and quantified by spectrophotometry. Single-stranded cDNA was amplified from 1 µg of total RNA using a qScript cDNA Synthesis Kit (Quanta, Beverly, MA, USA). Real-time quantitative PCR (qRT-PCR) analysis was performed with an automated Rotor-Gene system RG-3000A (Corbett Research, Sydney, Australia). The PCR reaction mix (20 µL) was composed of 10 µL of QPCR sybr master mix (Quanta, Beverly, MA, USA), 5 µL of diluted cDNA (each sample in triplicate) and a final concentration of 0.3 µM of each primer. Hypoxanthine guanine phosphoribosyl transferase (HPRT) primers were used as an internal standard. The following primers were utilized: mouse HPRT sense: 5′-GTC GTG ATT AGC GAT GAT GAA-3′, antisense: 5′-CTC CCA TCT CCT TCA TGA CAT C-3′; mouse Estrogen Receptor Alpha sense: 5′-GGG CTG ACT TCA CTT ACA TTT C-3′, antisense: 5′-GGA GCA TCT ACA GGA ACA CAG-3′; mouse TLR4 sense: 5′-ATG CAT GGA TCA GAA ACT CAG CAA-3′, antisense: 5′-AAA CTT CCT GGG GAA AAA CTC TGG-3′; mouse progesterone receptor sense: 5′-CCC ACA GGA GTT TGT CAA ACT C-3′, antisense: 5′-TAA CTT CAG ACA TCA TTT CCG G-3′; mouse PS2 sense: 5′-CTG CCC AGG AGA GAA ATG AG-3′, antisense: 5′-CAG GGT ATG AGG GTT CTC CA-3′; mouse TNFα sense: 5′-GCC TCT TCT CAT TCC TGC TTG-3′, antisense: 5′-CTG ATG AGA GGG AGG CCA TT-3′; human HPRT sense: 5′-GCT ATA AAT TCT TTG CTG ACC TGC T-3′, antisense: 5′-ATT ACT TTT ATG TCC CCT GTT GAC TG-3′; human Estrogen Receptor Alpha sense: 5′-TGA TGA AAG GTG GGA TAC GA-3′, antisense: 5′-AAG GTT GGC AGC TCT CAT GT-3′; human TLR4 Sense: 5′-ACC AAG AAC CTG GAC CTG AG-3′, antisense: 5′-TCT GGA TGG GGT TTC CTG TC-3′; human Aromatase Sense: 5′-CAC ATC CTC AAT ACC AGG TCC-3′, antisense: 5′-CAG AGA TCC AGA CTC GCA TG-3′; human TNFα Sense: 5′-CTG CCC CAA TCC CTT T-3′, antisense: 5′-CCC AAT TCT CTT TTT G-3′.

**Statistical analysis.** The results are presented as the mean ± SD unless otherwise stated. *p* values ≤ 0.05 were considered statistically significant. Statistical analysis of in vitro and in vivo data was performed by unpaired Student’s *t*-test, if not otherwise stated. All statistical tests were two-sided.

**Study approval.** Animal experiments were approved by the Institutional Animal Care Committee of the Hebrew University.

## Data Availability

All data generated or analyzed during this study are included in this article.

## Supplementary Materials

The following supporting information can be downloaded at: https://www.mdpi.com/article/10.3390/ijms27104338/s1.

## References

[1] Cani P.D., Amar J., Iglesias M.A., Poggi M., Knauf C., Bastelica D., Neyrinck A.M., Fava F., Tuohy K.M., Chabo C. (2007). Metabolic endotoxemia initiates obesity and insulin resistance. Diabetes.

[2] Cao J., Peng J., An H., He Q., Boronina T., Guo S., White M.F., Cole P.A., He L. (2017). Endotoxemia-mediated activation of acetyltransferase P300 impairs insulin signaling in obesity. Nat. Commun..

[3] Amar J., Burcelin R., Ruidavets J.B., Cani P.D., Fauvel J., Alessi M.C., Chamontin B., Ferrieres J. (2008). Energy intake is associated with endotoxemia in apparently healthy men. Am. J. Clin. Nutr..

[4] Boutagy N.E., McMillan R.P., Frisard M.I., Hulver M.W. (2016). Metabolic endotoxemia with obesity: Is it real and is it relevant?. Biochimie.

[5] Troseid M., Nestvold T.K., Rudi K., Thoresen H., Nielsen E.W., Lappegard K.T. (2013). Plasma lipopolysaccharide is closely associated with glycemic control and abdominal obesity: Evidence from bariatric surgery. Diabetes Care.

[6] Fuke N., Nagata N., Suganuma H., Ota T. (2019). Regulation of Gut Microbiota and Metabolic Endotoxemia with Dietary Factors. Nutrients.

[7] Gaber M., Wilson A.S., Millen A.E., Hovey K.M., LaMonte M.J., Wactawski-Wende J., Ochs-Balcom H.M., Cook K.L. (2024). Visceral adiposity in postmenopausal women is associated with a pro-inflammatory gut microbiome and immunogenic metabolic endotoxemia. Microbiome.

[8] Nahmias Blank D., Maimon O., Hermano E., Drai E., Chen O., Popovtzer A., Peretz T., Meirovitz A., Elkin M. (2025). Unraveling the Role of Metabolic Endotoxemia in Accelerating Breast Tumor Progression. Biomedicines.

[9] Thaiss C.A., Levy M., Grosheva I., Zheng D., Soffer E., Blacher E., Braverman S., Tengeler A.C., Barak O., Elazar M. (2018). Hyperglycemia drives intestinal barrier dysfunction and risk for enteric infection. Science.

[10] Neal M.D., Leaphart C., Levy R., Prince J., Billiar T.R., Watkins S., Li J., Cetin S., Ford H., Schreiber A. (2006). Enterocyte TLR4 mediates phagocytosis and translocation of bacteria across the intestinal barrier. J. Immunol..

[11] Vreugdenhil A.C., Rousseau C.H., Hartung T., Greve J.W., van’t Veer C., Buurman W.A. (2003). Lipopolysaccharide (LPS)-binding protein mediates LPS detoxification by chylomicrons. J. Immunol..

[12] Hersoug L.G., Moller P., Loft S. (2018). Role of microbiota-derived lipopolysaccharide in adipose tissue inflammation, adipocyte size and pyroptosis during obesity. Nutr. Res. Rev..

[13] Clemente-Postigo M., Oliva-Olivera W., Coin-Araguez L., Ramos-Molina B., Giraldez-Perez R.M., Lhamyani S., Alcaide-Torres J., Perez-Martinez P., El Bekay R., Cardona F. (2019). Metabolic endotoxemia promotes adipose dysfunction and inflammation in human obesity. Am. J. Physiol. Endocrinol. Metab..

[14] Neves A.L., Coelho J., Couto L., Leite-Moreira A., Roncon-Albuquerque R. (2013). Metabolic endotoxemia: A molecular link between obesity and cardiovascular risk. J. Mol. Endocrinol..

[15] Massier L., Bluher M., Kovacs P., Chakaroun R.M. (2021). Impaired Intestinal Barrier and Tissue Bacteria: Pathomechanisms for Metabolic Diseases. Front. Endocrinol..

[16] Jobe M., Agbla S.C., Todorcevic M., Darboe B., Danso E., de Barros J.P., Lagrost L., Karpe F., Prentice A.M. (2022). Possible mediators of metabolic endotoxemia in women with obesity and women with obesity-diabetes in The Gambia. Int. J. Obes..

[17] Manilla V., Di Tommaso N., Santopaolo F., Gasbarrini A., Ponziani F.R. (2023). Endotoxemia and Gastrointestinal Cancers: Insight into the Mechanisms Underlying a Dangerous Relationship. Microorganisms.

[18] Eibl G., Rozengurt E. (2021). Obesity and Pancreatic Cancer: Insight into Mechanisms. Cancers.

[19] NCI (2025). Obesity and Cancer Fact Sheet—NCI. https://www.cancer.gov/about-cancer/causes-prevention/risk/obesity/obesity-fact-sheet.

[20] Patel A.V., Patel K.S., Teras L.R. (2023). Excess body fatness and cancer risk: A summary of the epidemiologic evidence. Surg. Obes. Relat. Dis..

[21] Fink H., Langselius O., Vignat J., Rumgay H., Rehm J., Martinez R.X., Santero M., Lopez-Perez L., Inoue M., Zeng H. (2026). Global and regional cancer burden attributable to modifiable risk factors to inform prevention. Nat. Med..

[22] Dapito D.H., Mencin A., Gwak G.Y., Pradere J.P., Jang M.K., Mederacke I., Caviglia J.M., Khiabanian H., Adeyemi A., Bataller R. (2012). Promotion of hepatocellular carcinoma by the intestinal microbiota and TLR4. Cancer Cell.

[23] Kundu S.D., Lee C., Billips B.K., Habermacher G.M., Zhang Q., Liu V., Wong L.Y., Klumpp D.J., Thumbikat P. (2008). The toll-like receptor pathway: A novel mechanism of infection-induced carcinogenesis of prostate epithelial cells. Prostate.

[24] He Y., Ou Z., Chen X., Zu X., Liu L., Li Y., Cao Z., Chen M., Chen Z., Chen H. (2016). LPS/TLR4 Signaling Enhances TGF-beta Response Through Downregulating BAMBI During Prostatic Hyperplasia. Sci. Rep..

[25] Nahmias-Blank D., Maimon O., Meirovitz A., Sheva K., Peretz-Yablonski T., Elkin M. (2023). Excess body weight and postmenopausal breast cancer: Emerging molecular mechanisms and perspectives. Semin. Cancer Biol..

[26] Qureshi R., Picon-Ruiz M., Aurrekoetxea-Rodriguez I., Nunes de Paiva V., D’Amico M., Yoon H., Radhakrishnan R., Morata-Tarifa C., Ince T., Lippman M.E. (2020). The Major Pre- and Postmenopausal Estrogens Play Opposing Roles in Obesity-Driven Mammary Inflammation and Breast Cancer Development. Cell Metab..

[27] Le Naour A., Rossary A., Vasson M.P. (2020). EO771, is it a well-characterized cell line for mouse mammary cancer model? Limit and uncertainty. Cancer Med..

[28] Hiraga T., Ninomiya T. (2019). Establishment and characterization of a C57BL/6 mouse model of bone metastasis of breast cancer. J. Bone Miner. Metab..

[29] Gu J.W., Young E., Busby B., Covington J., Johnson J.W. (2009). Oral administration of pyrrolidine dithiocarbamate (PDTC) inhibits VEGF expression, tumor angiogenesis, and growth of breast cancer in female mice. Cancer Biol. Ther..

[30] Hawes M.L., Moody M.A., McCauley C.R., Huddleston A.G., Solanky M., Khosravi D.H., Patel A.R., Lynch R.M., Alahari S.K., Bunnell B.A. (2025). Oncogenic effects of ECM remodeling in obesity and breast cancer. Oncogene.

[31] Ritter A., Kreis N.N., Roth S., Friemel A., Safdar B.K., Hoock S.C., Wildner J.M., Allert R., Louwen F., Solbach C. (2023). Cancer-educated mammary adipose tissue-derived stromal/stem cells in obesity and breast cancer: Spatial regulation and function. J. Exp. Clin. Cancer Res..

[32] Hillers-Ziemer L.E., Kuziel G., Williams A.E., Moore B.N., Arendt L.M. (2022). Breast cancer microenvironment and obesity: Challenges for therapy. Cancer Metastasis Rev..

[33] Iyengar N.M., Gucalp A., Dannenberg A.J., Hudis C.A. (2016). Obesity and Cancer Mechanisms: Tumor Microenvironment and Inflammation. J. Clin. Oncol..

[34] Morris P.G., Hudis C.A., Giri D., Morrow M., Falcone D.J., Zhou X.K., Du B., Brogi E., Crawford C.B., Kopelovich L. (2011). Inflammation and increased aromatase expression occur in the breast tissue of obese women with breast cancer. Cancer Prev. Res..

[35] Singh A., Purohit A., Ghilchik M.W., Reed M.J. (1999). The regulation of aromatase activity in breast fibroblasts: The role of interleukin-6 and prostaglandin E2. Endocr. Relat. Cancer.

[36] Suzuki R., Orsini N., Saji S., Key T.J., Wolk A. (2009). Body weight and incidence of breast cancer defined by estrogen and progesterone receptor status—A meta-analysis. Int. J. Cancer.

[37] White A.J., Nichols H.B., Bradshaw P.T., Sandler D.P. (2015). Overall and central adiposity and breast cancer risk in the Sister Study. Cancer.

[38] Neuhouser M.L., Aragaki A.K., Prentice R.L., Manson J.E., Chlebowski R., Carty C.L., Ochs-Balcom H.M., Thomson C.A., Caan B.J., Tinker L.F. (2015). Overweight, Obesity, and Postmenopausal Invasive Breast Cancer Risk: A Secondary Analysis of the Women’s Health Initiative Randomized Clinical Trials. JAMA Oncol..

[39] Lauby-Secretan B., Scoccianti C., Loomis D., Grosse Y., Bianchini F., Straif K., International Agency for Research on Cancer Handbook Working Group (2016). Body Fatness and Cancer—Viewpoint of the IARC Working Group. N. Engl. J. Med..

[40] Sebastiani F., Cortesi L., Sant M., Lucarini V., Cirilli C., De Matteis E., Marchi I., Negri R., Gallo E., Federico M. (2016). Increased Incidence of Breast Cancer in Postmenopausal Women with High Body Mass Index at the Modena Screening Program. J. Breast Cancer.

[41] Pearson-Stuttard J., Zhou B., Kontis V., Bentham J., Gunter M.J., Ezzati M. (2018). Worldwide burden of cancer attributable to diabetes and high body-mass index: A comparative risk assessment. Lancet Diabetes Endocrinol..

[42] Lee K., Kruper L., Dieli-Conwright C.M., Mortimer J.E. (2019). The Impact of Obesity on Breast Cancer Diagnosis and Treatment. Curr. Oncol. Rep..

[43] Yang T.O., Cairns B.J., Pirie K., Green J., Beral V., Floud S., Reeves G.K. (2022). Body size in early life and the risk of postmenopausal breast cancer. BMC Cancer.

[44] Sho M., Qureshi R., Slingerland J. (2025). Oestrogen changes at menopause: Insights into obesity-associated breast risk and outcomes. Nat. Rev. Endocrinol..

[45] Fontvieille E., Jansana A., Peruchet-Noray L., Cordova R., Gan Q., Rinaldi S., Dossus L., Mahamat-Saleh Y., Gunter M.J., Heath A. (2025). Body mass index and breast cancer risk among postmenopausal women with and without cardiometabolic diseases: Findings from two prospective cohort studies in Europe. Cancer.

[46] Chamberlin T., Clack M., Silvers C., Kuziel G., Thompson V., Johnson H., Arendt L.M. (2020). Targeting Obesity-Induced Macrophages during Preneoplastic Growth Promotes Mammary Epithelial Stem/Progenitor Activity, DNA Damage, and Tumor Formation. Cancer Res..

[47] Nahmias Blank D., Hermano E., Sonnenblick A., Maimon O., Rubinstein A.M., Drai E., Maly B., Vlodavsky I., Popovtzer A., Peretz T. (2022). Macrophages Upregulate Estrogen Receptor Expression in the Model of Obesity-Associated Breast Carcinoma. Cells.

[48] Bulun S.E., Chen D., Moy I., Brooks D.C., Zhao H. (2012). Aromatase, breast cancer and obesity: A complex interaction. Trends Endocrinol. Metab..

[49] Zhao Y., Nichols J.E., Valdez R., Mendelson C.R., Simpson E.R. (1996). Tumor necrosis factor-alpha stimulates aromatase gene expression in human adipose stromal cells through use of an activating protein-1 binding site upstream of promoter 1.4. Mol. Endocrinol..

[50] Purohit A., Reed M.J. (2002). Regulation of estrogen synthesis in postmenopausal women. Steroids.

[51] Simpson E.R., Brown K.A. (2013). Obesity and breast cancer: Role of inflammation and aromatase. J. Mol. Endocrinol..

[52] Zahid H., Simpson E.R., Brown K.A. (2016). Inflammation, dysregulated metabolism and aromatase in obesity and breast cancer. Curr. Opin. Pharmacol..

[53] Kim M., Lee C., Park J. (2022). Extracellular matrix remodeling facilitates obesity-associated cancer progression. Trends Cell Biol..

[54] Brown K.A., Iyengar N.M., Zhou X.K., Gucalp A., Subbaramaiah K., Wang H., Giri D.D., Morrow M., Falcone D.J., Wendel N.K. (2017). Menopause Is a Determinant of Breast Aromatase Expression and Its Associations with BMI, Inflammation, and Systemic Markers. J. Clin. Endocrinol. Metab..

[55] Lumeng C.N., Saltiel A.R. (2011). Inflammatory links between obesity and metabolic disease. J. Clin. Investig..

[56] Nickell W.B., Skelton J. (2005). Breast fat and fallacies: More than 100 years of anatomical fantasy. J. Hum. Lact..

[57] Simpson E.R. (2003). Sources of estrogen and their importance. J. Steroid Biochem. Mol. Biol..

[58] Maitra U., Deng H., Glaros T., Baker B., Capelluto D.G., Li Z., Li L. (2012). Molecular mechanisms responsible for the selective and low-grade induction of proinflammatory mediators in murine macrophages by lipopolysaccharide. J. Immunol..

[59] Leenen P.J., de Bruijn M.F., Voerman J.S., Campbell P.A., van Ewijk W. (1994). Markers of mouse macrophage development detected by monoclonal antibodies. J. Immunol. Methods.

[60] MacKinnon A.C., Farnworth S.L., Hodkinson P.S., Henderson N.C., Atkinson K.M., Leffler H., Nilsson U.J., Haslett C., Forbes S.J., Sethi T. (2008). Regulation of alternative macrophage activation by galectin-3. J. Immunol..

[61] Tanaka Y., Ohno T., Kadonaga T., Kidokoro Y., Wakahara M., Nosaka K., Sakabe T., Suzuki Y., Nakamura H., Umekita Y. (2021). Podoplanin expression in cancer-associated fibroblasts predicts unfavorable prognosis in node-negative breast cancer patients with hormone receptor-positive/HER2—Negative subtype. Breast Cancer.

[62] Cui M., Dong H., Duan W., Wang X., Liu Y., Shi L., Zhang B. (2024). The relationship between cancer associated fibroblasts biomarkers and prognosis of breast cancer: A systematic review and meta-analysis. PeerJ.

[63] Arendt L.M., McCready J., Keller P.J., Baker D.D., Naber S.P., Seewaldt V., Kuperwasser C. (2013). Obesity promotes breast cancer by CCL2-mediated macrophage recruitment and angiogenesis. Cancer Res..

[64] Hermano E., Goldberg R., Rubinstein A.M., Sonnenblick A., Maly B., Nahmias D., Li J.P., Bakker M.A.H., van der Vlag J., Vlodavsky I. (2019). Heparanase Accelerates Obesity-Associated Breast Cancer Progression. Cancer Res..

[65] Incio J., Tam J., Rahbari N.N., Suboj P., McManus D.T., Chin S.M., Vardam T.D., Batista A., Babykutty S., Jung K. (2016). PlGF/VEGFR-1 Signaling Promotes Macrophage Polarization and Accelerated Tumor Progression in Obesity. Clin. Cancer Res..

[66] Ning C., Xie B., Zhang L., Li C., Shan W., Yang B., Luo X., Gu C., He Q., Jin H. (2016). Infiltrating Macrophages Induce ERalpha Expression through an IL17A-mediated Epigenetic Mechanism to Sensitize Endometrial Cancer Cells to Estrogen. Cancer Res..

[67] Hammond M.E., Hayes D.F., Dowsett M., Allred D.C., Hagerty K.L., Badve S., Fitzgibbons P.L., Francis G., Goldstein N.S., Hayes M. (2010). American Society of Clinical Oncology/College of American Pathologists guideline recommendations for immunohistochemical testing of estrogen and progesterone receptors in breast cancer. Arch. Pathol. Lab. Med..

[68] Brown K.A. (2021). Metabolic pathways in obesity-related breast cancer. Nat. Rev. Endocrinol..

[69] Argolo D.F., Hudis C.A., Iyengar N.M. (2018). The Impact of Obesity on Breast Cancer. Curr. Oncol. Rep..

[70] McTiernan A., Wu L., Chen C., Chlebowski R., Mossavar-Rahmani Y., Modugno F., Perri M.G., Stanczyk F.Z., Van Horn L., Wang C.Y. (2006). Relation of BMI and physical activity to sex hormones in postmenopausal women. Obesity.

[71] Madigan M.P., Troisi R., Potischman N., Dorgan J.F., Brinton L.A., Hoover R.N. (1998). Serum hormone levels in relation to reproductive and lifestyle factors in postmenopausal women (United States). Cancer Causes Control.

[72] Lukanova A., Lundin E., Zeleniuch-Jacquotte A., Muti P., Mure A., Rinaldi S., Dossus L., Micheli A., Arslan A., Lenner P. (2004). Body mass index, circulating levels of sex-steroid hormones, IGF-I and IGF-binding protein-3: A cross-sectional study in healthy women. Eur. J. Endocrinol..

[73] Bezemer I.D., Rinaldi S., Dossus L., Gils C.H., Peeters P.H., Noord P.A., Bueno-de-Mesquita H.B., Johnsen S.P., Overvad K., Olsen A. (2005). C-peptide, IGF-I, sex-steroid hormones and adiposity: A cross-sectional study in healthy women within the European Prospective Investigation into Cancer and Nutrition (EPIC). Cancer Causes Control.

[74] Morgan M.M., Arendt L.M., Alarid E.T., Beebe D.J., Johnson B.P. (2019). Mammary adipose stromal cells derived from obese women reduce sensitivity to the aromatase inhibitor anastrazole in an organotypic breast model. FASEB J..

[75] Samarajeewa N.U., Yang F., Docanto M.M., Sakurai M., McNamara K.M., Sasano H., Fox S.B., Simpson E.R., Brown K.A. (2013). HIF-1alpha stimulates aromatase expression driven by prostaglandin E2 in breast adipose stroma. Breast Cancer Res..

[76] Santen R.J., Simpson E. (2019). History of Estrogen: Its Purification, Structure, Synthesis, Biologic Actions, and Clinical Implications. Endocrinology.

[77] Bhattacharyya S., Midwood K.S., Yin H., Varga J. (2017). Toll-Like Receptor-4 Signaling Drives Persistent Fibroblast Activation and Prevents Fibrosis Resolution in Scleroderma. Adv. Wound. Care.

[78] Bhattacharyya S., Wang W., Qin W., Cheng K., Coulup S., Chavez S., Jiang S., Raparia K., De Almeida L.M.V., Stehlik C. (2018). TLR4-dependent fibroblast activation drives persistent organ fibrosis in skin and lung. JCI Insight.

[79] Apte R.N. (1995). Mechanisms of cytokine production by fibroblasts-implications for normal connective tissue homeostasis and pathological conditions. Folia Microbiol..

[80] Faust H.J., Cheng T.Y., Korsunsky I., Watts G.F.M., Gal-Oz S.T., Trim W.V., Kongthong S., Jonsson A.H., Simmons D.P., Zhang F. (2024). Adipocyte associated glucocorticoid signaling regulates normal fibroblast function which is lost in inflammatory arthritis. Nat. Commun..

[81] Nazari B., Rice L.M., Stifano G., Barron A.M., Wang Y.M., Korndorf T., Lee J., Bhawan J., Lafyatis R., Browning J.L. (2016). Altered Dermal Fibroblasts in Systemic Sclerosis Display Podoplanin and CD90. Am. J. Pathol..

[82] Bulun S.E., Sharda G., Rink J., Sharma S., Simpson E.R. (1996). Distribution of aromatase P450 transcripts and adipose fibroblasts in the human breast. J. Clin. Endocrinol. Metab..

[83] Pagano C., di Zazzo E., Avilia G., Savarese B., Navarra G., Proto M.C., Fiore D., Rienzo M., Gazzerro P., Laezza C. (2022). Advances in “adiponcosis”: Insights in the inner mechanisms at the base of adipose and tumour tissues interplay. Int. J. Cancer.

[84] Argyrakopoulou G., Dalamaga M., Spyrou N., Kokkinos A. (2021). Gender Differences in Obesity-Related Cancers. Curr. Obes. Rep..

[85] Simati S., Kokkinos A., Dalamaga M., Argyrakopoulou G. (2023). Obesity Paradox: Fact or Fiction?. Curr. Obes. Rep..

[86] Kang C., LeRoith D., Gallagher E.J. (2018). Diabetes, Obesity, and Breast Cancer. Endocrinology.

[87] Papakonstantinou E., Piperigkou Z., Karamanos N.K., Zolota V. (2022). Altered Adipokine Expression in Tumor Microenvironment Promotes Development of Triple Negative Breast Cancer. Cancers.

[88] Wang X., Lin W.J., Izumi K., Jiang Q., Lai K.P., Xu D., Fang L.Y., Lu T., Li L., Xia S. (2012). Increased infiltrated macrophages in benign prostatic hyperplasia (BPH): Role of stromal androgen receptor in macrophage-induced prostate stromal cell proliferation. J. Biol. Chem..

[89] Majorini M.T., Cancila V., Rigoni A., Botti L., Dugo M., Triulzi T., De Cecco L., Fontanella E., Jachetti E., Tagliabue E. (2020). Infiltrating Mast Cell-Mediated Stimulation of Estrogen Receptor Activity in Breast Cancer Cells Promotes the Luminal Phenotype. Cancer Res..

[90] Ewertz M., Jensen M.B., Gunnarsdottir K.A., Hojris I., Jakobsen E.H., Nielsen D., Stenbygaard L.E., Tange U.B., Cold S. (2011). Effect of obesity on prognosis after early-stage breast cancer. J. Clin. Oncol..

[91] Gnant M., Pfeiler G., Stoger H., Mlineritsch B., Fitzal F., Balic M., Kwasny W., Seifert M., Stierer M., Dubsky P. (2013). The predictive impact of body mass index on the efficacy of extended adjuvant endocrine treatment with anastrozole in postmenopausal patients with breast cancer: An analysis of the randomised ABCSG-6a trial. Br. J. Cancer.

[92] Petrelli F., Cortellini A., Indini A., Tomasello G., Ghidini M., Nigro O., Salati M., Dottorini L., Iaculli A., Varricchio A. (2021). Association of Obesity with Survival Outcomes in Patients with Cancer: A Systematic Review and Meta-analysis. JAMA Netw. Open.

[93] Sestak I., Distler W., Forbes J.F., Dowsett M., Howell A., Cuzick J. (2010). Effect of body mass index on recurrences in tamoxifen and anastrozole treated women: An exploratory analysis from the ATAC trial. J. Clin. Oncol..

[94] Alzet Technical Information Manual. https://www.alzet.com/products/alzet_pumps/performance/.

[95] Hermano E., Carlotti F., Abecassis A., Meirovitz A., Rubinstein A.M., Li J.P., Vlodavsky I., Rabelink T.J., Elkin M. (2021). Dichotomic role of heparanase in a murine model of metabolic syndrome. Cell Mol. Life Sci..

[96] Lerner I., Hermano E., Zcharia E., Rodkin D., Bulvik R., Doviner V., Rubinstein A.M., Ishai-Michaeli R., Atzmon R., Sherman Y. (2011). Heparanase powers a chronic inflammatory circuit that promotes colitis-associated tumorigenesis in mice. J. Clin. Investig..

[97] Orimo A., Gupta P.B., Sgroi D.C., Arenzana-Seisdedos F., Delaunay T., Naeem R., Carey V.J., Richardson A.L., Weinberg R.A. (2005). Stromal fibroblasts present in invasive human breast carcinomas promote tumor growth and angiogenesis through elevated SDF-1/CXCL12 secretion. Cell.

[98] Zawieracz K., Eckert M.A. (2022). Isolation of Normal and Cancer-Associated Fibroblasts. Methods Mol. Biol..

[99] Cheang M.C., Treaba D.O., Speers C.H., Olivotto I.A., Bajdik C.D., Chia S.K., Goldstein L.C., Gelmon K.A., Huntsman D., Gilks C.B. (2006). Immunohistochemical detection using the new rabbit monoclonal antibody SP1 of estrogen receptor in breast cancer is superior to mouse monoclonal antibody 1D5 in predicting survival. J. Clin. Oncol..

