# Supplementary material for: Metabolic Endotoxemia Amplifies Estrogen Signaling Through Stromal Crosstalk in Obesity-Associated Breast Cancer
Source: Int J Mol Sci. 2026 May 13;27(10):4338. doi: 10.3390/ijms27104338 (PMC13207763; doi:10.3390/ijms27104338)
Supplement: Supplementary file 1 [file ijms-27-04338-s001.zip › ijms-4261718-supplementary.pdf]

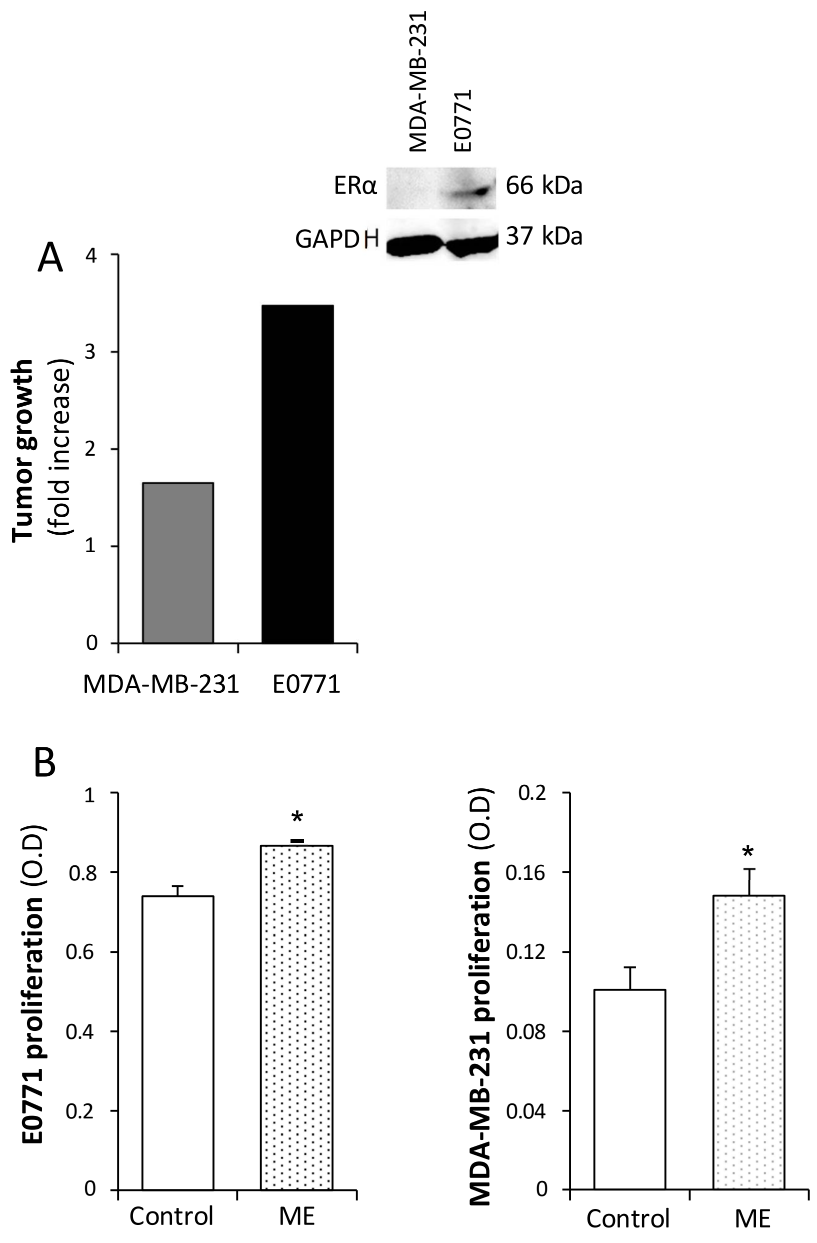

Figure S1. Effect of metabolic endotoxemia conditions on ER+ E0771 and ER- MDA- MB-231 breast carcinoma growth *in vivo* (A) and *in vitro* (B). A. Mice were implanted sc. with the Alzet osmotic minipump (as described in Methods) filled with either saline (**Control**) or endotoxin (to infuse 300 mg per Kg per day, resulting in chronic experimental metabolic endotoxemia, **ME**). Human BC cells MDA- MB-231 or murine E0771 BC cells were injected orthotopically 3 days after pump implantation, as described in Methods. Tumor growth was monitored for 15 (E0771) or 18 (MDA-MB-231) days. The bar graph represents a fold increase in the volume of MDA-MB-231 (grey bar) and E0771 (black bar) tumors grown in endotoxin-infused vs. control mice (n= 5 mice per experimental group). **Inset**: ERα protein levels in MDA-MB-231 and E0771 cells were determined by immunoblotting. MCF7 cells were used as a positive control for ERα expression. **B.** E0771 cells (**left**) and MDA-MB-231 cells (**right**) were cultured *in vitro* either in the absence (control, empty bars) or presence (dotted bars) of 0.1 ng/ml of endotoxin. Cell growth was assessed by MTS assay (in pentaplicates). Data are the mean±SD; p values were determined by Student's t-test. *<0.02.

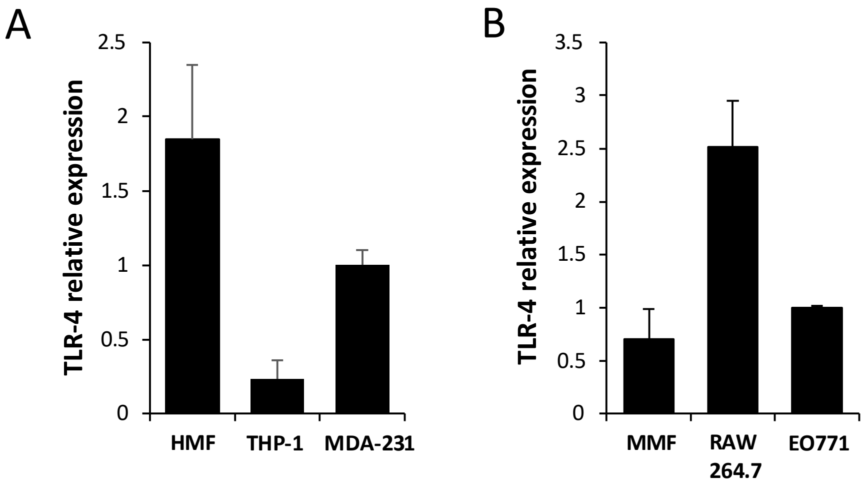

Figure S2. Expression levels of TLR4 in adipose fibroblasts, monocytes and BC cells of human (A) and murine (B) origin. A. Levels of TLR4 expression in human mammary adipose fibroblasts (HMF), human monocytic cell line THP-1 and human BC cell line MDA-231 were analyzed by qRT-PCR.

B. Levels of TLR4 expression in mouse mammary adipose fibroblasts (MMF), mouse monocytic cell line RAW 264.7 and mouse BC cell line E0771 were analyzed by qRT-PCR. Data are the mean±SD

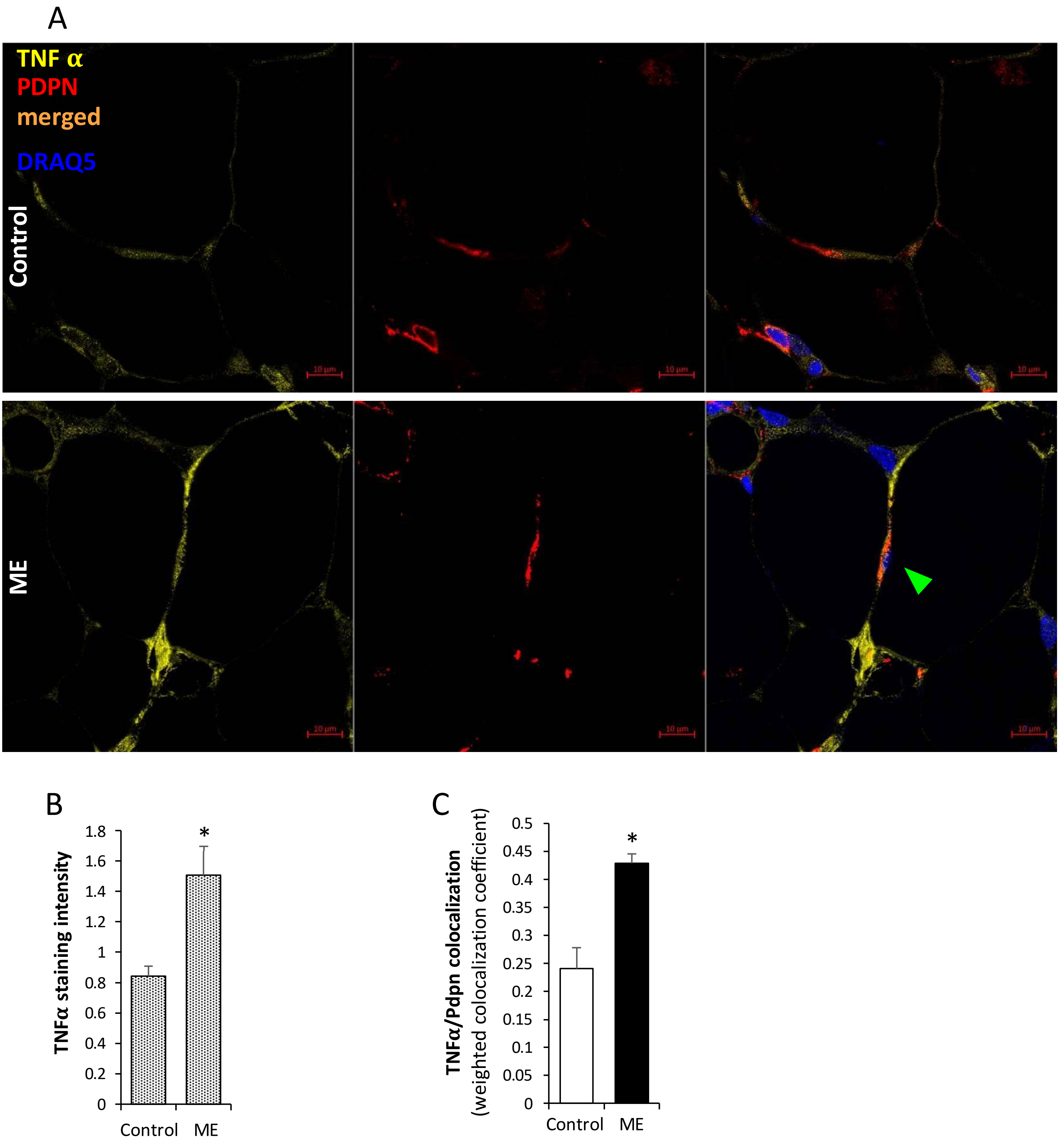

Figure S3. Effect of ME conditions on TNFα expression by adipose fibroblasts *in vivo.*

A. Sections of mouse adipose tissue samples derived from saline-infused (**control**) and endotoxin-infused (**ME**) mice (n≥3) were harvested 18 days after pump implantation and processed for double immunofluorescent analysis using anti- TNFα (yellow) and anti-Pdpn (red) antibodies. Green arrowhead indicates TNFα^{+}/Pdpn^{+} fibroblast. Cell nuclei were counterstained with DRAQ5 (blue). Photographs are representative of **Control** (upper panels) and **ME** (lower panels) samples. Scale bar = 10 µm. **B**. Staining intensity of TNFα (**B**) and Pdpn was quantified based on at least four sections from 3 mice per condition. using Zen software (Carl Zeiss) per 0.01 mm^{2} microscopic field. **C.** Quantification of the degree of association between TNFα and Pdpn staining in mouse adipose derived from **Control (empty bar)** and endotoxin-infused (**ME, filled bar**) mice (n≥3) was performed using colocalization tool of Zen software. Bar graph shows the weighted correlation coefficient values for TNFα /Pdpn colocalization, Data are the mean±SE; two-sided Student's t test *p<0.03).
